# Sex differences in surgical treatment of pleural empyema: an exploratory retrospective comparison of video-assisted thoracoscopic surgery and thoracotomy

**DOI:** 10.1186/s13019-025-03741-y

**Published:** 2025-12-09

**Authors:** Josef Yayan, Marcus Krüger, Christian Biancosino

**Affiliations:** 1 Department of Internal Medicine, Division of Pulmonary, Allergy, and Sleep Medicine, Helios University Hospital Wuppertal, Witten/Herdecke University, Heusnerstr. 40, Wuppertal, 42283 Germany; 2 Department of Thoracic Surgery, Helios University Hospital Wuppertal, Witten/Herdecke University, Witten, Germany; 3 Department of Thoracic Surgery, Martha-Maria Hospital Halle-Dölau, Halle, Germany

**Keywords:** Abscess, Pleural empyema, Sex differences, Thoracic surgery, Thoracotomy, Video-assisted thoracoscopic surgery

## Abstract

**Background:**

Pleural empyema is a severe infectious condition that requires timely surgical management. While video-assisted thoracoscopic surgery (VATS) and open thoracotomy are both established treatment modalities, limited data exist regarding sex-specific differences in clinical presentation, intraoperative findings, and outcomes. Previous studies have seldom explored whether biological sex influences local disease extent, such as abscess formation, or short-term perioperative outcomes.

**Methods:**

In this retrospective single-center study, adult patients who underwent surgery for pleural empyema between December 1, 2019, and May 31, 2024, were analyzed. Patients were grouped according surgical approach (VATS or thoracotomy) and stratified by sex. Demographic, clinical, laboratory, and intraoperative data were collected. Comparisons between male and female patients were conducted using Fisher’s exact test and unpaired t-tests, with a significance level set to* P* < 0.05. Postoperative outcomes were assessed until hospital discharge; no long-term follow-up was available.

**Results:**

A total of 103 patients were included (73 men, 30 women); 19 underwent VATS and 84 thoracotomy. In the VATS group, no significant sex-related differences were found in age, laboratory values, complications, or intraoperative findings. In the thoracotomy group, a significantly higher rate of intraoperatively detected abscesses was observed in female patients compared to male patients (78.3% vs. 45.9%, *P* = 0.016). No other statistically significant differences were found between sexes in hemoglobin, leukocyte counts, CRP, ICU admission, or mortality.

**Conclusion:**

A higher prevalence of intraoperative abscesses was observed in female patients undergoing thoracotomy for pleural empyema, despite a lower burden of systemic comorbidities. Otherwise, outcomes and perioperative parameters were similar between sexes in both surgical groups. These findings are exploratory and hypothesis-generating and should be interpreted with caution. Larger prospective studies with balanced sex representation, standardized staging, and long-term follow-up are warranted to clarify potential sex-related variations in disease manifestation and surgical outcomes. The relatively small female cohort and limited VATS subgroup reduce statistical power and restrict generalizability.

## Introduction

Pleural empyema represents a serious and potentially life-threatening condition characterized by the accumulation of purulent fluid within the pleural space [1]. Despite advances in diagnostic and therapeutic approaches, the optimal management of empyema remains a matter of debate, particularly with regard to surgical technique and patient-related factors [2]. Video-assisted thoracoscopic surgery (VATS) and open thoracotomy are both established procedures for the surgical treatment of advanced empyema, offering varying advantages in terms of invasiveness, postoperative recovery, and complications [3, 4].

In recent years, increasing attention has been directed toward biological sex differences in the presentation and progression of thoracic infectious diseases [5]. Biological sex is known to influence immune response, symptom perception, and treatment outcomes in various infectious and inflammatory conditions [6, 7]. For example, females often exhibit a stronger inflammatory response with greater cytokine release compared to males, which may lead to faster clearance of infection but could also contribute to differences in local disease manifestations such as abscess formation [6–9]. However, in the context of pleural empyema, data on sex-based differences remain scarce. Most existing studies focus on general prognostic factors such as age, comorbidity burden, and microbiological findings, but fail to differentiate between male and female patients in terms of clinical presentation or outcomes [8, 9].

As sex may affect treatment response and recovery, understanding potential differences between male and female patients are clinically relevant. Of particular interest is whether sex influences intraoperative findings, such as abscesses that may remain undetected preoperatively, or whether the surgical approach itself interacts with sex-related disease patterns. It also remains uncertain if such differences translate into variations in complication rates, intensive care unit (ICU) admission, or perioperative mortality.

This study aims to address this knowledge gap by comparing male and female patients with pleural empyema who underwent either VATS or thoracotomy. The objective was to analyze sex-specific differences in demographic and clinical characteristics, intraoperative findings, and early postoperative outcomes. Given the limited number of women and the relatively small VATS subgroup, this study is exploratory and aims to generate hypotheses rather than provide definitive, practice-changing conclusions. Identifying such differences may contribute to more individualized treatment strategies and improved surgical decision-making in the management of pleural empyema [10].

## Materials and methods

### Study design and objective

This retrospective observational study was designed to evaluate sex-specific differences in the clinical presentation, intraoperative characteristics, and early postoperative outcomes of patients undergoing surgical treatment for pleural empyema. The primary objective was to compare male and female patients and generate exploratory hypotheses regarding potential sex-specific patterns, rather than to establish causal relationships.

### Study setting and patient population

The study was conducted at the Department of Thoracic Surgery at Helios University Hospital Wuppertal, a tertiary academic institution affiliated with Witten/Herdecke University. The analysis included all consecutive adult patients aged 18 years or older who underwent surgery for pleural empyema between December 1, 2019, and May 31, 2024.

### Eligibility criteria

Patients were included if they had a confirmed diagnosis of pleural empyema based on clinical, radiological, or intraoperative findings and received surgical management through either video-assisted thoracoscopic surgery (VATS) or open thoracotomy. Only patients with complete documentation of relevant clinical and laboratory parameters were considered. Patients who underwent surgery for indications other than empyema or who had incomplete datasets were excluded from the analysis. No matching or propensity score adjustment was performed; thus, all comparisons are unadjusted and exploratory.

### Surgical approach

The choice between VATS and thoracotomy was based on the stage and extent of the empyema, intraoperative feasibility, and the surgeon’s judgment. VATS was generally preferred in early fibrinopurulent stages, while thoracotomy was performed in more advanced or organized empyema, in patients with extensive pleural peels, or in cases where minimally invasive access was not sufficient. In some cases, procedures initially started as VATS were converted to thoracotomy. Patients who required conversion were analyzed in the thoracotomy group.

### Definition of abscess

An intraoperative abscess was defined as a macroscopically visible encapsulated collection of pus within the pleural cavity, as documented in the operative report. These abscesses were often not identifiable on preoperative imaging and were diagnosed intraoperatively.

### Data collection

Clinical data were collected retrospectively from the hospital’s electronic medical record system. Demographic parameters included age, sex, and smoking status. Documented comorbidities encompassed cardiovascular, pulmonary, renal, and metabolic conditions. Laboratory values recorded prior to surgery included hemoglobin concentration, leukocyte count, serum creatinine, and C-reactive protein levels. Intraoperative findings, such as presence of an abscess, detection of pathogens, and operative duration, were documented. Postoperative data included admission to intensive care, need for transfusion, occurrence of complications, and in-hospital mortality. Follow-up data beyond hospital discharge were not systematically available and thus long-term outcomes, recurrence rates, and survival could not be assessed.

### Sex comparison

The primary analysis focused on the comparison between male and female patients. Male and female patients were analyzed separately for each surgical approach (VATS and thoracotomy) to assess potential sex-specific differences. This subgroup approach was chosen because the stage of empyema and the feasibility of minimally invasive surgery strongly influenced whether patients underwent VATS or thoracotomy. Clinical and laboratory variables, intraoperative findings, and outcome parameters were compared between the sexes.

### Statistical analysis

All data were analyzed using descriptive and inferential statistical methods. Continuous variables are presented as means ± standard deviation, and categorical variables as absolute numbers and percentages. Statistical comparisons between male and female patients were performed using the unpaired Student’s t-test for continuous variables and Fisher’s exact test for categorical variables. Statistical significance was defined as a two-sided P value less than 0.05. Confidence intervals were not calculated due to the limited sample size; therefore, results should be interpreted as exploratory. Statistical analyses were conducted using R, version 4.3.2 (R Foundation for Statistical Computing, Vienna, Austria), validated statistical software widely applied in biomedical research.

## Results

**Fig. 1:**
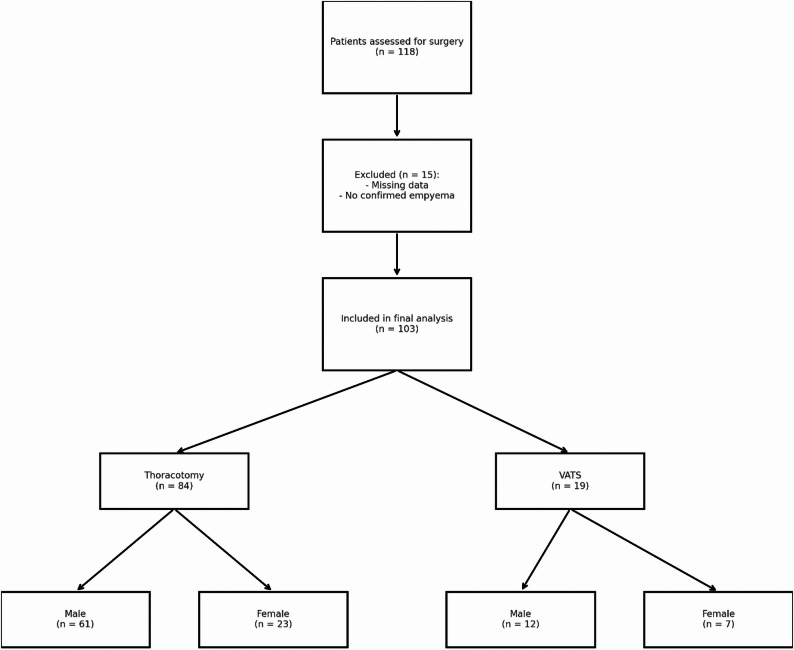
Flowchart illustrating patient inclusion by surgical approach and sex

This retrospective study included 103 consecutive adult patients who underwent surgical treatment for pleural empyema between December 1, 2019, and May 31, 2024, at the Department of Thoracic Surgery, Helios University Hospital Wuppertal, a tertiary academic center of Witten/Herdecke University. Of these, 19 patients (12 men, 7 women) underwent video-assisted thoracoscopic surgery (VATS). In addition, 84 patients (61 men, 23 women), including three who required intraoperative conversion from VATS due to advanced disease, were treated with thoracotomy (Fig. 1). This corresponds to a conversion rate of 15.8% within the VATS group and 2.9% across the entire cohort.

A sex-based comparison within the VATS cohort (Table 1) revealed no statistically significant differences in demographic, clinical, or laboratory characteristics. The mean age was 60.4 ± 20.2 years in men and 50.3 ± 24.8 years in women (*P* = 0.3795). Laboratory parameters including hemoglobin (12.1 ± 1.6 vs. 11.8 ± 1.4 g/dL; *P* = 0.6809), creatinine (0.9 ± 0.5 vs. 0.8 ± 0.2 mg/dL; *P* = 0.6991), leukocytes (11,011.7 ± 4379.5 vs. 13,362.9 ± 9825.4/µL; *P* = 0.5668), and CRP (8.2 ± 8.1 vs. 10.0 ± 11.8 mg/dL; *P* = 0.7267) were comparable between sexes. No significant differences were found in complication rates, ICU admissions, or operative duration. Microbiological detection of pathogens was more frequent in women (57.1%) than in men (16.7%), although not statistically significant (*P* = 0.1287). No transfusions were required in this group.

**Fig. 2:**
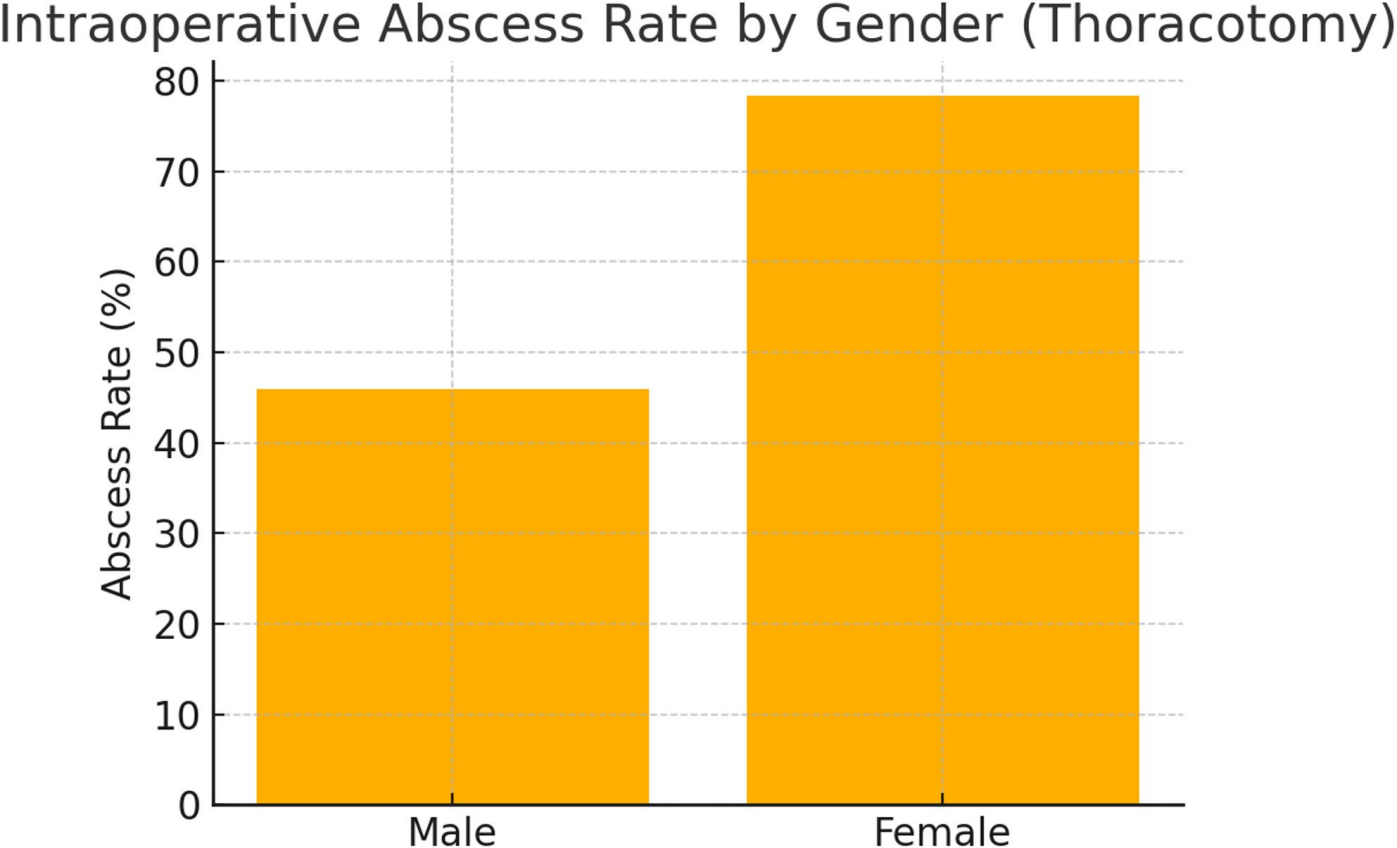
Sex comparison of intraoperative abscess rates in thoracotomy

In contrast, the thoracotomy cohort (Table 2) showed a significant difference in the incidence of intraoperatively detected abscesses, which were present in 78.3% of women versus 45.9% of men (*P* = 0.016; Fig. 2). These abscesses were often not identifiable preoperatively and diagnosed only during exploration. Other variables, including age (61.7 ± 15.1 vs. 54.9 ± 16.1 years; *P* = 0.085), hemoglobin (11.4 ± 1.9 vs. 10.9 ± 1.5 g/dL; *P* = 0.225), leukocytes (13,090.0 ± 5651.0 vs. 13,528.7 ± 5795.7/µL; *P* = 0.757), CRP (12.9 ± 10.5 vs. 17.0 ± 16.0 mg/dL; *P* = 0.266), and creatinine (0.9 ± 0.3 vs. 1.1 ± 1.8 mg/dL; *P* = 0.618), did not differ significantly. ICU admission and transfusion rates were likewise similar.

When comparing both surgical modalities, outcomes in the VATS group were relatively consistent without sex-specific differences. In the thoracotomy group, however, the significantly higher abscess rate in women suggests a possible sex-related variation in local disease manifestation. This interpretation should be considered exploratory, given the retrospective design and limited sample size. Other clinical variables remained largely consistent across sexes in both groups.

In the analysis of ASA classification by sex, a significant difference in distribution was observed. The majority of men were classified as ASA III (70.5%), indicating a higher burden of systemic disease and surgical risk, compared to 52.2% of women. Conversely, a larger proportion of women were ASA II (34.8% vs. 24.6% in men). ASA I was rare in both groups, but more frequent among women (13.0% vs. 3.3%). These findings suggest that women may have a lower prevalence of severe systemic disease compared to men.

**Fig. 3:**
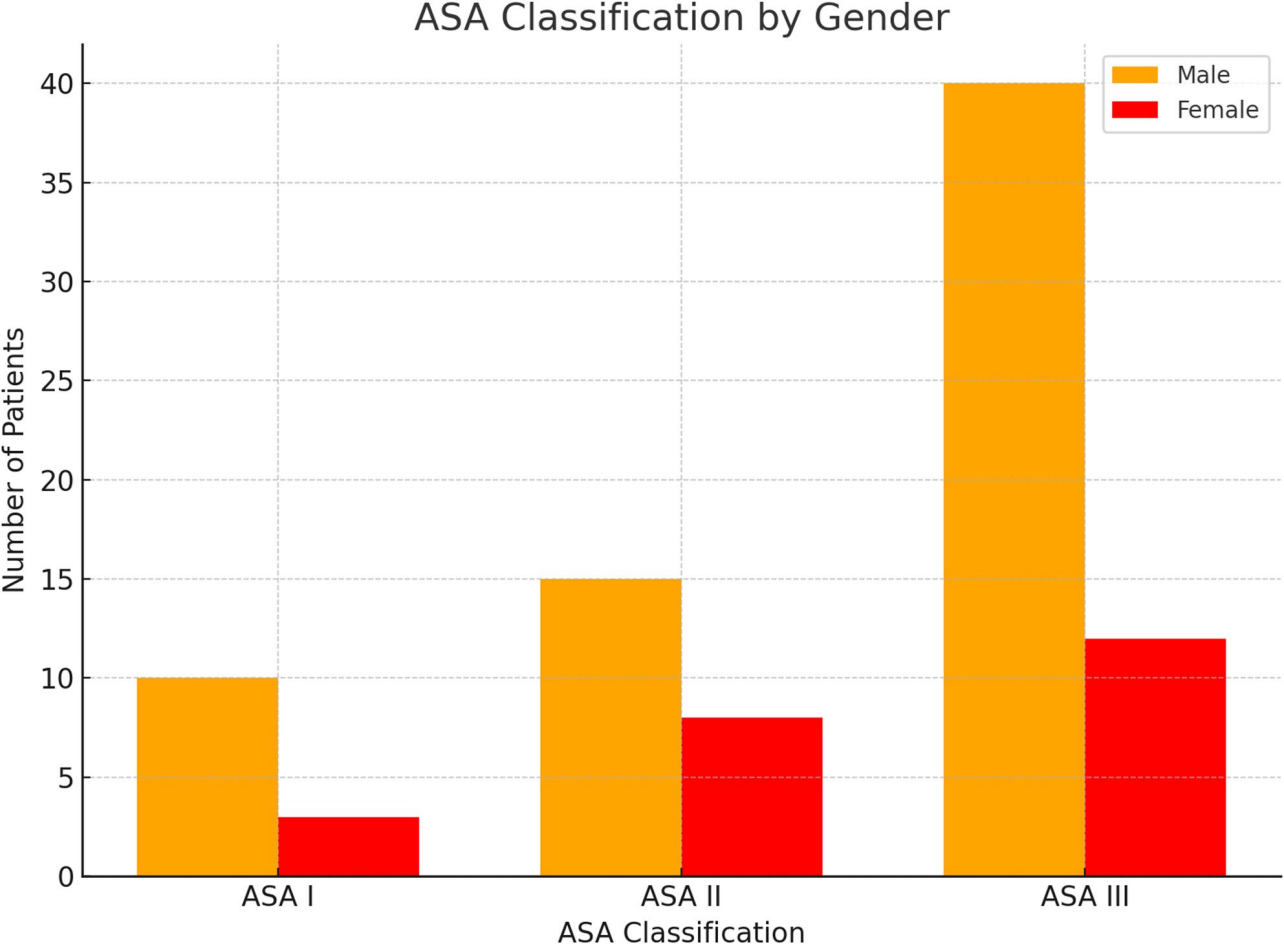
ASA classification by sex, indicating a higher proportion of ASA class III in male patients compared to female patients

Regarding intraoperative conditions, the rate of abscess formation was markedly higher in women undergoing thoracotomy (78.3% vs. 45.9% in men; Fig. 3). These abscesses were documented intraoperatively and not systematically identifiable preoperatively. This difference may reflect sex-related variations in immune response, disease progression, or timing of presentation. However, given the retrospective design and the relatively small female sample size, these observations should be interpreted with caution. Rather than supporting sex-tailored surgical strategies at present, the findings are exploratory and emphasize the need for prospective studies to investigate potential biological and clinical mechanisms underlying these differences.

**Table 1:** Sex-based comparison of clinical and demographic characteristics in patients undergoing video-assisted thoracoscopic surgery (VATS). Continuous variables are presented as mean ± standard deviation (SD); categorical variables as numbers (percentages). *P* values were calculated using fisher’s exact test (categorical variables) and unpaired t-test (continuous variables); *P* < 0.05 was considered statistically significant. “n.a.” indicates not applicable

Variable	Male patients *N* = 12 (%)	Female patients *N* = 7 (%)	*P* value
Age (mean ± SD)	60.4 ± 20.2	50.3 ± 24.8	0.3795
Nicotine	4 (33.3)	3 (42.9)	1.0
Blood thinning	3 (25.0)	1 (14.3)	1.0
ASA I	1 (8.3)	2 (28.6)	0.5232
ASA II	3 (25.0)	2 (28.6)	1.0
ASA III	8 (66.7)	3 (42.9)	0.3765
Right-sided empyema	8 (66.7)	4 (57.1)	1.0
Left-sided empyema	4 (33.3)	3 (42.9)	1.0
Complication	1 (8.3)	1 (14.3)	1.0
Death	3 (25.0)	0	0.2632
Pneumonia	1 (8.3)	1(14.3)	1.0
Abscess	3 (25.0)	2 (28.6)	1.0
Hemoglobin (mean ± SD) (g/dL)	12.1 ± 1.6	11.8 ± 1.4	0.6809
Creatinine preoperative (mean ± SD) (mg/dL)	0.9 ± 0.5	0.8 ± 0.2	0.6991
Leukocytes preoperative (mean ± SD) (10^9/µL)	11011.7 ± 4379.5	13362.9 ± 9825.4	0.5668
C-reactive protein (CRP) (mean ± SD) (mg/dL)	8.2 ± 8.1	10.0 ± 11.8	0.7267
Microorganism intraoperative	2 (16.7)	4 (57.1)	0.1287
Surgery duration (minutes, mean ± SD)	104.8 ± 35.6	90.9 ± 72.2	0.6468
Blood transfusion	0	0	n.a.
Intensive Care Unit (ICU)	7 (58.3)	4 (57.1)	1.0
Arterial hypertension	2 (16.7)	0	0.5088
Chronic kidney insufficiency	0	2 (28.6)	0.1228
Hypertensive heart disease	2 (16.7)	2 (28.6)	0.6027
Diabetes	3 (25.0)	0	0.2632
Obesity	1 (8.3)	0	1.0
Chronic obstructive pulmonary disease (COPD)	1 (8.3)	0	1.0
Coronary heart disease	1 (8.3)	2 (28.6)	0.5232

ASA: American Society of Anesthesiologists physical status classification. SD: Standard Deviation

**Table 2:** Sex-based comparison of clinical and demographic characteristics in patients undergoing thoracotomy. Continuous variables are presented as mean ± standard deviation (SD); categorical variables as numbers (percentages). *P* values were calculated using fisher’s exact test (categorical variables) and unpaired t-test (continuous variables); *P* < 0.05 was considered statistically significant. “n.a.” indicates not applicable

Variable	Male patients *N* = 61 (%)	Female patients *N* = 23 (%)	*P* value
Age (mean ± SD)	61.7 ± 15.1	54.9 ± 16.1	0.085
Nicotine	25 (41.0)	10 (43.5)	1.0
Blood thinning	9 (14.8)	4 (17.4)	1.0
ASA I	2 (3.3)	3 (13.0)	0.242
ASA II	15 (24.6)	8 (34.8)	0.509
ASA III	43 (70.5)	12 (52.2)	0.188
Right-sided empyema	33 (54.1)	13 (56.5)	1.0
Left-sided empyema	28 (45.9)	10 (43.5)	1.0
Complication	31 (50.8)	12 (52.2)	1.0
Death	0	0	n.a.
Pneumonia	5 (8.2)	1 (4.3)	0.892
Abscess	28 (45.9)	18 (78.3)	**0.016**
Hemoglobin (mean ± SD) (g/dL)	11.4 ± 1.9	10.9 ± 1.5	0.225
Creatinine preoperative (mean ± SD) (mg/dL)	0.9 ± 0.3	1.1 ± 1.8	0.618
Leukocytes preoperative (mean ± SD) (10^9/µL)	13090.0 ± 5651.0	13528.7 ± 5795.7	0.757
C-reactive protein (CRP) (mean ± SD) (mg/dL)	12.9 ± 10.5	17.0 ± 16.0	0.266
Microorganism intraoperative	21 (34.4)	8 (34.8)	1.0
Surgery duration (minutes, mean ± SD)	117.5 ± 30.5	113.4 ± 36.5	0.637
Blood transfusion	17 (27.9)	7 (30.4)	1.0
Intensive Care Unit (ICU)	54 (88.5)	19 (82.6)	0.723
Arterial hypertension	20 (32.8)	6 (26.1)	0.743
Chronic kidney insufficiency	2 (3.3)	3 (13.0)	0.242
Hypertensive heart disease	7 (11.5)	4 (17.4)	0.723
Diabetes	8 (13.1)	2 (8.7)	0.857
Obesity	3 (4.9)	1 (4.3)	1.0
Chronic obstructive pulmonary disease (COPD)	7 (11.5)	3 (13.0)	1.0
Coronary heart disease	4 (6.6)	1 (4.3)	1.0

ASA: American Society of Anesthesiologists physical status classification. SD: Standard Deviation. Significant *P *values are in bold

## Discussion

This study examined sex-specific differences in patients undergoing surgical treatment for pleural empyema, comparing outcomes between male and female patients treated either by video-assisted thoracoscopic surgery (VATS) or open thoracotomy. While most clinical and laboratory parameters were comparable between sexes, a significantly higher rate of intraoperative abscess formation was observed among female patients undergoing thoracotomy. These abscesses were defined in this study as encapsulated purulent collections, often not identifiable on preoperative imaging, underscoring the limitations of radiological staging and the relevance of intraoperative exploration.

Previous literature on pleural empyema has mainly focused on age, comorbidities, microbiological etiology, and surgical technique as prognostic factors, while sex-specific aspects have rarely been addressed [11, 12]. However, evidence from infectious disease research shows that sex differences influence immune responses and clinical presentation. Women generally mount stronger innate and adaptive immune responses, including enhanced cytokine release and neutrophil activation, which may accelerate systemic pathogen clearance but paradoxically promote localized inflammatory abscess formation [13, 14]. This dual effect may help explain why female patients in our cohort exhibited a higher prevalence of intraoperative abscesses despite a lower proportion classified as ASA III.

Similar paradoxes have been described in other infectious contexts, where women experience less systemic deterioration but more localized disease manifestations. In our cohort, men more frequently had higher ASA classifications, reflecting a greater comorbidity burden, while women more often showed intraoperative abscesses. This discordance suggests that systemic disease severity and local disease progression may be modulated differently by sex.

No significant sex-specific differences were observed in mortality, ICU admission, transfusion requirements, or laboratory parameters, this is consistent with previous reports indicating no sex-based disadvantage in survival among surgically treated empyema patients [15]. From a surgical perspective, these results are reassuring, suggesting that both thoracotomy and VATS remain effective treatment options regardless of sex, at least with respect to short-term outcomes.

Our results did not show significant sex-related differences in the VATS group, which may reflect the smaller sample size or the less invasive character of VATS. Moreover, the surgical approach itself is closely linked to empyema stage: VATS is typically chosen in earlier fibrinopurulent phases, whereas thoracotomy is reserved for more advanced or organized disease [11, 12]. The higher abscess rate in women undergoing thoracotomy may therefore partly reflect stage-related differences, although staging was not systematically assessed in this cohort. However, the lower abscess prevalence in men undergoing the same procedure indicates that stage alone cannot fully explain the observed sex disparity. In our cohort, three patients required conversion from VATS to thoracotomy due to intraoperatively detected abscesses or extensive adhesions, and were analyzed in the thoracotomy group. This reinforces the importance of intraoperative findings in guiding surgical decision-making.

Clinically, these findings should not be interpreted as supporting sex-specific surgical strategies, such as a preference use of thoracotomy in women. Instead, they are exploratory and hypothesis-generating, highlighting the potential relevance of sex as a biological variable in empyema research. Future multicenter prospective studies with larger, more balanced cohorts and standardized staging are needed to confirm these observations and clarify their implications for individualized treatment.

### Limitations

This study has several limitations. First, its retrospective design inherently precludes causal inference and is subject to potential selection and information biases.

Second, the single-center setting may restrict the generalizability of the findings to other institutions or populations with different clinical and demographic characteristics.

Third, the relatively small sample size, particularly among female patients in the VATS group, reduces statistical power and increases the risk of type II error.

Fourth, the absence of long-term follow-up data prevents assessment of sex-specific outcomes beyond the perioperative period, such as recurrence, long-term morbidity, or quality of life.

Finally, unmeasured confounders such as socioeconomic status, hormonal influences, or delays in presentation may have contributed to the observed sex differences. Despite these limitations, the study provides valuable exploratory insights into sex-associated patterns in surgically treated pleural empyema and underscores the need for prospective, multicenter trials with larger, balanced cohorts and standardized follow-up.

## Conclusion

This retrospective study identified a significantly higher rate of intraoperative abscess formation in women undergoing thoracotomy for pleural empyema, whereas other clinical and perioperative variables were largely comparable between sexes across both surgical approaches. No sex-specific differences were observed in mortality, ICU admission, or laboratory parameters. These findings are exploratory and hypothesis-generating, and they do not justify sex-based modification of surgical strategies at present. Instead, they suggest that sex may influence local disease manifestation in advanced stages requiring open surgery.

Future multicenter prospective studies with larger and more balanced cohorts, standardized staging, and long-term follow-up are needed to validate these results and clarify the potential role of sex-specific factors in the surgical management of pleural empyema.

## Data Availability

All data generated or analyzed during this study are included in this published article.

## Declarations

### Trial registration

Not applicable. This is a retrospective study and was not registered as a clinical trial.
